# Simultaneous microextraction of pesticides from wastewater using optimized μSPEed and μQuEChERS techniques for food contamination analysis

**DOI:** 10.1016/j.heliyon.2023.e16742

**Published:** 2023-05-26

**Authors:** Laura García-Cansino, María Ángeles García, María Luisa Marina, José S. Câmara, Jorge A.M. Pereira

**Affiliations:** a Universidad de Alcalá, Departamento de Química Analítica, Química Física e Ingeniería Química, Ctra. Madrid-Barcelona Km. 33.600, 28871, Alcalá de Henares, Madrid, Spain; b CQM-UMa, Centro de Química da Madeira, Campus Universitário da Penteada, 9000-390, Funchal, Portugal; c Universidad de Alcalá, Instituto de Investigación Química Andrés M. del Río, Ctra. Madrid-Barcelona Km. 33.600, 28871, Alcalá de Henares, Madrid, Spain; d Faculdade de Ciências Exatas e Engenharia da Universidade da Madeira, Campus Universitário da Penteada, 9000-390, Funchal, Portugal

**Keywords:** Pesticides, Microextraction, μSPEed, μQuEChERS, Wastewater, Sample preparation, Ultrahigh-performance liquid chromatography, Residue analysis, Environmental monitoring, EU legislation, Maximum residue levels, Validation

## Abstract

Food contamination with pesticides poses significant risks to consumer safety and undermines confidence in food supply chains. Detecting pesticides in food samples is a challenging task that requires efficient extraction techniques. This study aims to compare and validate two microextraction techniques, μSPEed and μQuEChERS-dSPE, for the simultaneous extraction of eight pesticides (paraquat, thiabendazole, asulam, picloram, ametryn, atrazine, linuron, and cymoxanil) from wastewater samples. A good analytical performance was obtained for both methodologies, with selectivity, linearity in the range 0.5–150 mg L^−1^ with coefficients of determination up to 0.9979, limits of detection (LODs) and limits of quantification (LOQs) ranging from 0.02 to 0.05 mg L^−1^ and from 0.06 to 0.17 mg L^−1^, respectively, precision below 14.7 mg L^−1^, and recoveries from wastewater samples in the range of 66.1–99.9%. The developed methodologies are simpler, faster, and require less sample and solvent volumes than conventional methodologies, having a lower impact on the environment. Nevertheless, the μSPEed approach was found to be more efficient, easier to perform, and with a higher greener profile. This study highlights the potential of microextraction techniques for the analysis of pesticide residues in food and environmental samples. Overall, it presents a fast and efficient method for the analysis of pesticides in wastewater samples, which can be useful for monitoring and controlling pesticide contamination in the environment.

## Highlights

• Microextraction techniques were optimized for simultaneous pesticides extraction.
• μSPEed and μQuEChERS-dSPE are more advantageous than classical extraction procedures.
• μSPEed and μQuEChERS-dSPE were coupled to a fast UHPLC analysis with PDA detection.
• Methodologies validated for the analysis of selected pesticides in wastewater.

## 1 Introduction

Pesticides are commonly used to protect crops from various threats and to increase yields in shorter time. However, incorrect or excessive use can result in high levels of pesticide residues in food products [[1], [2], [3], [4]], which can have negative effects on human health and the environment [[5], [6], [7], [8]]. Sometimes, even with proper use, the presence of pesticide residues in food and environmental samples (such as vegetables, fruits, water, and soil samples) is unavoidable [9,10], necessitating additional measures to mitigate contamination. Therefore, it is crucial to control the presence of pesticide residues in food matrices for consumer safety and human health. Pesticides are broadly classified based on their target organisms. Herbicides, for example, are used to control the growth of unwanted plant species that compete with a particular crop species, affecting their growth. In this group, we can find pesticides such as paraquat (pQ) [11], asulam (ASU) [12], picloram (PIC) [13], ametryn (AME) [14], atrazine (ATR) [15], and linuron (LIN) [16], among others, some of which are of great concern for human and animal health and the environment. ASU, for instance, can be an important water pollutant due to its formulation with sodium salt [12]. PIC (4-amino-3,5,6-trichloropyridine-2-carboxylic acid) is another herbicide that can remain active in the soil for quite some time, bound to organic matter and clay particles. However, if the soil consists of little organic matter or clay, picloram is displaced with water and may leach into the groundwater [13]. Fungicides are another group of pesticides widely used for different purposes, namely, to kill fungus parasites that colonize crop species. This is the case with thiabendazole (THIA, 4-(1H-benzimidazol-2-yl)-1,3-thiazole), a post-harvesting pesticide used to preserve citrus fruits during transport and storage [17] and cymoxanil (CYM, (1E)-2-(ethylcarbamoylamino)-N-methoxy-2-oxoethanimidoyl cyanide) used against grape downy mildew [18]. Table 1 aggregates relevant information on selected pesticides, their classification, toxicity and authorized maximum residue levels (MRLs) in foodstuffs according to the legislation adopted by the European Union [19]. Due to the low concentrations at which pesticide residues are found in real samples, efficient sample treatment procedures for the extraction or pre-concentration of the target analytes are often necessary [20].

**Table 1:** Classification of selected pesticides according to their hazard level, and maximum residue levels (MRLs) established in the European Union.

#	*Class*^a^	*MRL*^b^ (mg kg^−1^)
pQ	II	0.02–0.05
THIA	III	0.01–7.00^c^
ASU	III	0.02–0.10
PIC	U	0.01^d^
AME	II	–
ATR	III	0.05–0.10
LIN	III	0.01–0.05
CYM	II	0.01–0.10^e^

a *Class*: II, moderately hazardous; III, slightly hazardous; U, unlikely to present acute hazard in normal use.

b *MRL*: maximum residue level.

c All data are low except citrus fruits, pome fruits, bananas, mangoes, and papayas.

d High levels were observed for products of animal origin.

e 0.9 value was observed for spinaches.

Solid phase extraction (SPE) has been one of the most widely used sample extraction procedures in analytical chemistry in recent decades. However, in recent years, several SPE configurations have been developed to reduce the volume of reagents and samples, decreasing their environmental impact, and making the procedure more environmentally friendly. Microextraction by packed sorbent (MEPS) employs a sorbent tightly packed inside a cartridge (BIN) through which the liquid extract is withdrawn up and down. The target analytes are concomitantly retained in the sorbent and eluted with a suitable solvent. This technique can be performed manually, semi-automatically or automatically [[21], [22], [23]]. An improved configuration of MEPS, micro solid phase extraction (μSPEed), has been recently proposed to further improve MEPS potential. This new format has different advantages such as its pressure-driven one-way direct flow through the sorbent bed. This means that sample aspiration, unlike MEPS, which is bidirectional, is performed solely by the vacuum generated by the syringe plunger and always in a single direction. In addition, the use of smaller sorbent particles (3 μm or smaller compared to the 50 μm diameter used in MEPS) increases the contact surface area, allowing for more efficient extraction of the target analytes [24]. This innovative μSPEed approach can be operated under different configurations (reviewed in Ref. [24]). The semi-automated version, controlled by an electronic syringe, allows greater control of the experimental conditions and consequent optimization of the extraction efficiency. Overall, the μSPEed configuration allows a great level of miniaturization and sample preconcentration. In turn, μQuEChERS is a simple and inexpensive method that is miniaturized from the popular salting-out QuEChERS procedure. This extraction procedure, proposed by Anastassiades et al. [25] is based on the dispersion of partition salts in a solution containing an organic solvent (salting-out effect). The target analytes are isolated in the organic extract, cleaned up by dispersive SPE, and the extract is dried and resuspended in the final solvent. This analytical procedure is simple, fast, and does not require any sophisticated laboratory equipment or glass labware, nor great expertise. For this reason, it was rapidly adopted and applied to many other applications beyond the original pesticide extraction [25,26]. Meanwhile, despite its simplicity, QuEChERS stills uses large volumes of solvents and samples and so its miniaturization (μQuEChERS) would make the procedure even more interesting and environmentally friendly, as shown by Porto-Figueira et al. [27] and Casado et al. [28].

This study aims to compare and validate two microextraction techniques, μSPEed and μQuEChERS-dSPE, for the simultaneous extraction of eight pesticides from wastewater samples, followed by a fast chromatographic analysis with a photodiode array. The developed methodologies are simpler, faster, and require less sample and solvent volumes than conventional methodologies, with a lower impact on the environment. The analytical performance of both methods was evaluated and compared, and the μSPEed approach was found to be more efficient. The study highlights the potential of microextraction techniques for the analysis of pesticide residues in food and environmental samples.

## 2 Materials and methods

### 2.1 Reagents and materials

HPLC grade acetonitrile (ACN), methanol (MeOH), sodium hydroxide (NaOH), and ethyl acetate (EtAc) were from Fischer Scientific (Loughborough, UK). Formic acid (FA), chloride acid (HCl, 37%), and two buffered salts (sodium chloride - NaCl), anhydrous magnesium sulphate - MgSO_4_)), were obtained from Panreac Química (Barcelona, Spain). The other two buffered salts (disodium hydrogen citrate sesquihydrate (C_6_H_8_Na_2_O_8_), and trisodium citrate dihydrate (C_6_H_5_Na_3_O_7_·2H_2_O)) were from Sigma-Aldrich (St. Louis, MO, USA). Ultrapure water (H_2_O) was from Milli-Q ultrapure water purification system (Millipore, Bedford, MA, USA) and used for preparing the UHPLC mobile phase and other aqueous solutions. The dSPE clean-up DisQuE™ tubes with secondary amine (PSA) and MgSO_4_ were supplied by CHROMAtific UG (Heidenrod, Germany).

The pesticides used in this work, pQ (MW 257.16 g mol^−1^, ≥98%), THIA (MW 205.25 g mol^−1^, ≥98%), ASU (MW 230.24 g mol^−1^, ≥95%), PIC (MW 241.46 g mol^−1^, ≥98%), AME (MW 227.33 g mol^−1^, ≥98%), ATR (MW 215.68 g mol^−1^, ≥98%), LIN (MW 249.09 g mol^−1^, ≥98%), and CYM (MW 198.18 g mol^−1^, ≥98%), were purchased from Sigma-Aldrich (St. Louis, MO, USA). All reagents and other chemicals were of the highest analytical quality available.

The digiVol® syringe and the μSPEed® cartridges (octadecyl silica (C18), porous PS/DVB reversed phase (DVB-RP), unmodified silica (Si), polyfluoroalkyl substances (PFAS), butyl silica (C4), octyl silica (C8), aminopropyl silane sorbent (APS), porous PS/DVB normal phase (DVB-NP), PS/DVB cationic exchange (SCX), and PS/DVB anionic exchange (SAX)) to perform the microextractions by μSPEed technique were kindly supplied by EPREP (Mulgrave, Victoria, Australia).

### 2.2 Samples and standard solutions

All standard solutions were prepared at 1000 mg L^−1^ (except for pQ which was prepared at 500 mg L^−1^), using different solvents: MeOH for ATR, LIN, PIC, THIA, ACN for CYM and ACN (50%, v/v) for pQ, ASU, AME. When necessary, different dilutions at lower concentrations were prepared from previous standard solutions using the same solvents. Standard solutions were stored at 5 °C in the dark, except for the validation studies performed in this work. In this case, the working dilutions containing the selected pesticides were prepared daily by dilution with MeOH. The wastewater samples were provided by the canteen of the University of Madeira. Briefly, the water used to wash the fruits and vegetables served in the canteen was collected, aliquoted in 50 mL falcon tubes, centrifuged (5 min, 4000 rpm), the supernatant collected, and stored at −20 °C. Before use, the sample was thawed and filtered (0.2 μm PTFE filters).

### 2.3 Extraction procedures

#### 2.3.1 μSPEed

**Fig. 1:**
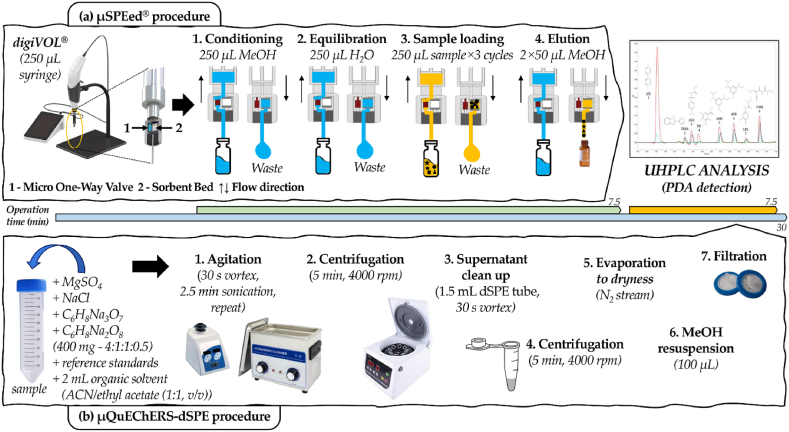
Overview of the **(a)** μSPEed and **(b)** μQuEChERS-dSPE optimized extraction procedures employed in this work.

The μSPEed method was performed with the electronic automatic syringe digiVol® X-change® (250 μL needle). Under the optimized conditions, the sorbent cartridge was conditioned with 250 μL of MeOH, equilibrated with 250 μL of H_2_O, three cycles of 250 μL of the sample were loaded with a flow rate of 1000 μL min^−1^, and finally, the elution was made with two cycles of 50 μL of MeOH with a flow rate of 500 μL min^−1^. The μSPEed extraction procedures take less than 7.5 min by sample. Each time a new cartridge was used, cartridge conditioning and equilibration steps were done twice. A simplified overview of the experimental procedure is depicted in Fig. 1a.

#### 2.3.2 μQuEChERS-dSPE

The μQuEChERS-dSPE extraction carried out in this work was adapted from the experimental optimization described by Porto-Figueira et al. [27] and Casado et al. [28] with minor adjustments. Accordingly, the mixture of the partition salts (MgSO_4_, NaCl, C_6_H_5_Na_3_O_7_.2H_2_O, and C_6_H_9_NaO_8_, 4:1:1:0.5) was downsized to 400 mg, spin vortex to homogenise, added to 500 μL sample with containing selected amounts of pesticides standards (doping) when necessary, spin vortex and add 2 mL ethyl acetate:ACN (1:1, v:v), containing 0.1% formic acid, as the organic extraction solvent. The mixture was subjected to two cycles of 2.5 min ultrasound bath (BRANSON 2510, 100W) followed by 30 s vortex and centrifuged 5 min at 4000 rpm. Then, the supernatant was subjected to dSPE with 150 mg of magnesium sulphate and 25 mg of PSA, by vortexing for 30 s, and centrifuged at 5 min 4000 rpm. The new supernatant was transferred to a glass tube and evaporated under a nitrogen stream. Finally, the dried extract was resuspended in 100 μL MeOH, filtered and injected (2 μL) into the UPLC, as described in Fig. 1b. Overall, the μQuEChERS-dSPE procedure takes around 30 min to complete, although several samples can be simultaneously extracted, depending on the equipment capacities.

### 2.4 UPLC-PDA analysis and operating conditions

A Waters Ultra-High Pressure Liquid Chromatographic Acquity system (UPLC, Acquity H-Class) (Milford, MA, USA) equipped with a column heater, an Acquity sample manager (SM), a degassing system, a Water Acquity quaternary solvent manager (QSM), and a photodiode array (PDA) detector (UPLC Acquity H-Class, Milford, MA, USA), was employed to analyse the selected pesticides in this work. An Acquity HSS T3 analytical column (2.1 mm × 100 mm, 1.8 μm particle size) set at 40 °C was chosen to carry out the assays. A gradient composed of 0.1% FA (solvent A) and ACN (solvent B) was employed as the mobile phase, being successively optimized to achieve the best chromatographic separation in the shortest time possible for the eight pesticides selected in this work. Accordingly, the best gradient conditions found were as follows: 95 to 70% A (1.75 min), 70 to 20% A (3 min), 20–95% A (0.25 min), 95% A (2.5 min), total running time of 7.5 min at a flow rate of 250 μL min^−1^. The system was re-equilibrated for 2 min with 95% A between injections. Each target analyte was detected with UV detection wavelength at its maximum absorbance and its identification was confirmed by comparison of the retention time and respective PDA spectra of pure standard solutions. The data obtained were processed using Empower 2 software (Waters).

### 2.5 Analytical validation

Both microextraction techniques were evaluated and compared in terms of selectivity, linearity, limits of detection (LODs), limits of quantification (LOQs), matrix effect, trueness on real samples, and precision (intra-day and inter-day). Selectivity was evaluated by the absence of interferences at the same retention times in the chromatogram of the target analytes. Linearity was assessed at seven different concentration levels within the linear dynamic ranges (LDR) limits indicated in Table 2, using standard solutions that were analyzed upon the microextraction procedures described above. The concentration ranges were chosen according to the sensitivity of the UHPLC-PDA and the respective LDR and MRLs. Linearity for each analyte was obtained by plotting the peak areas versus the respective concentrations. LODs (lowest concentration at which each analyte is identifiable above system noise) and LOQs (lowest concentration at which each analyte is quantifiable and can be measured with precision and trueness) were calculated using the standard deviation of the intercept divided by the slope of each calibration curve multiplied by 3.29 and 10, respectively. The matrix effect (ME, expressed in percentage (%)) was evaluated according to the following equation:

$$ME\left(\%\right)=\frac{A}{B}x\;100$$

being *A,* the mean of the peak areas corresponding to the analytes in the standard solution and *B,* the mean of the peak areas corresponding to the analytes in the wastewater sample, both after microextraction procedures.

**Table 2:** Comparison of the analytical figures between both proposed methodologies, μSPEed/UPLC-PDA and μQuEChERS-dSPE/UPLC-PDA.

#	*λ*_max_^a^	RT^b^	MT^c^	LDR^d^	Equation	r^2^	LODs^e^	LOQs^f^	ME^g^
pQ	268	1.4	*μSPEed*	5.0–150.0	y = 9439 × – 45614	0.9903	0.05	0.17	96.1
			*μQuEChERS*	–	–	–	–	–	–
THIA	301	2.7	*μSPEed*	0.5–75.0	y = 8195 × – 17141	0.9938	0.04	0.13	99.9
			*μQuEChERS*	1.0–200.0	y = 10075 × + 26656	0.9932	0.03	0.10	94.0
ASU	269	3.1	*μSPEed*	0.5–50.0	y = 4061 × – 1086	0.9943	0.04	0.11	80.1
			*μQuEChERS*	1.0–200.0	y = 17221 × – 64	0.9960	0.03	0.08	91.8
PIC	224	3.5	*μSPEed*	0.5–75.0	y = 2823 × – 788	0.9979	0.02	0.06	91.0
			*μQuEChERS*	–	–	–	–	–	–
AME	222	4.6	*μSPEed*	0.5–50.0	y = 18284 × + 25876	0.9914	0.05	0.16	89.7
			*μQuEChERS*	1.0–200.0	y = 17830 × + 64741	0.9934	0.03	0.10	90.5
ATR	220	5.4	*μSPEed*	0.5–75.0	y = 28743 × – 35779	0.9902	0.04	0.11	101.3
			*μQuEChERS*	1.0–200.0	y = 25432 × + 94933	0.9921	0.04	0.11	92.5
LIN	250	6.0	*μSPEed*	0.5–50.0	y = 7821 × – 3937	0.9961	0.03	0.08	76.0
			*μQuEChERS*	1.0–200.0	y = 6967 × + 20080	0.9921	0.04	0.11	88.3
CYM	242	6.8	*μSPEed*	0.5–75.0	y = 40137 × – 46769	0.9917	0.04	0.11	85.4
			*μQuEChERS*	1.0–200.0	y = 32752 × + 111582	0.9931	0.04	0.11	93.1

a λ_max_: Maximum Wavelength (nm).

b RT: Retention Time (min).

c Microextraction Technique.

d LDR: Linear Dynamic Range, in mg L^−1^.

e LODs: Limit of detection (mg L^−1^).

f LOQs: Limit of quantification (mg L^−1^).

g ME: Matrix Effect (%).

Trueness was expressed as recovery percentage (%) and calculated according to the following equation:

$$Recovery\left(\%\right)=\frac{Concentration\;Fortified\;Sample-Concentration\;Sample}{Theoretical\;Conc.}\;x\;100$$

where *Concentration Fortified Sample* is the concentration of pesticide present in a spiked water sample with standard solutions at medium level, *Concentration Sample* is the concentration of pesticide present in a non-spiked water sample, and *Theoretical Concentration* is the theoretical concentration to be added to the sample. Finally, precision was assessed as intra- and inter-day precision at three concentration levels (low, medium, and high level) and expressed as a percentage of relative standard deviation (% RSD). Intra-day was calculated by analysing six replicates in triplicate on the same day (repeatability), while inter-day was calculated by analysing three replicates in triplicate on three consecutive days (reproducibility).

## 3 Results and discussion

### 3.1 μSPEed optimization

**Fig. 2:**
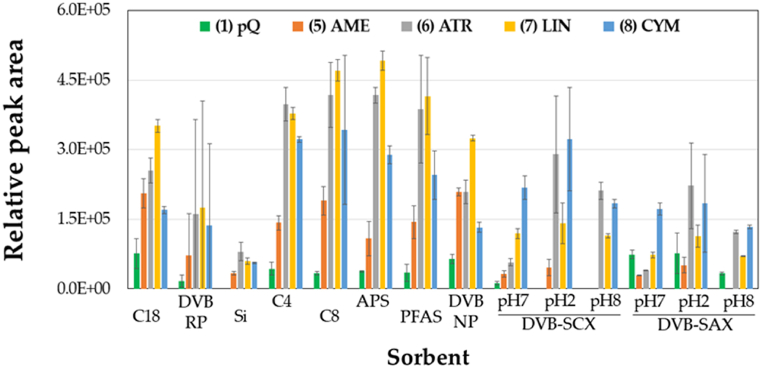
Assay of the sorbent effect on the extraction of the selected analytes employing the μSPEed procedure.

To achieve maximum efficiency in the μSPEed procedure, we follow a univariate optimization. Based on our previous experience with μSPEed extraction [23,29], the starting conditions involve conditioning the μSPEed cartridge with 250 μL of MeOH, equilibrating with 250 μL of H_2_O, loading 250 μL of sample (composed of a mixture of selected pesticide standard solutions at 25 mg L^−1^), washing with 250 μL of H_2_O, and eluting with 100 μL of MeOH. The flow rate for the different steps is 1000 μL min^−1^, except for the sample loading step, which is 500 μL min^−1^. Ten different cartridges, C18, DVB-RP, Si, PFAS, C4, C8, APS, DVB-NP, SCX, and SAX, were tested to find the sorbent that provided the best extraction efficiency. The ion exchange cartridges, SCX and SAX, were also assayed under acidic (pH 2.0) and basic (pH 8.0) conditions to verify the pH influence on the extraction procedure. According to the results obtained (Fig. 2), C18 was chosen as the best cartridge due to the overall analyte recovery values, i.e., considering all analytes and not each one independently.

After selecting the best sorbent, each step of the μSPEed procedure was revised for further optimization (see Fig. 1 for details). Every discarded solution in the different steps was analyzed to verify the efficiency of the target analytes retention during sample loading (step 3 in Fig. 1a); the level of interferents retained that were washed (omitted step between step 3 and step 4 in Fig. 1a), the efficiency of the elution step (step 3 in Fig. 1a) and the eventual existence of carryover by analysing the methanol loading in the first step of the following extraction (step 1 in Fig. 1a). Overall, the best extraction conditions were achieved by adding two additional sample loading cycles of 250 μL (total sample loading volume of 750 μL) and eluting twice with 50 μL of MeOH instead of a single elution of 100 μL (unlike the single elution, a two cycle elution allowed full recovery of the retained pesticides, data not shown). The washing step between sample loading and elution was also tested with a wastewater sample spiked with all the selected pesticides at 25 mg L^−1^, and no interferents were retained or washed in this step (data not shown). Therefore, the washing step in the μSPEed procedure was skipped. A detailed scheme of the μSPEed experimental layout is presented in Fig. 1a.

### 3.2 μQuEChERS-dSPE

QuEChERS is the reference extraction methodology for pesticide analysis, so it would be very relevant to compare its efficiency with the μSPEed approach proposed in this work. Taking into consideration that we have previously optimized a μQuEChERS-dSPE protocol involving fewer salts and solvents, therefore producing fewer wastes, and having a lower environmental impact ([27,28]), a μQuEChERS-dSPE procedure was also used in this work as a term of comparison with the μSPEed approach. The respective protocol is described in detail in Material and Methods (section 2.3.2) and a graphical overview of the procedure is provided in Fig. 1b.

### 3.3 Optimization of the chromatographic separation

**Fig. 3:**
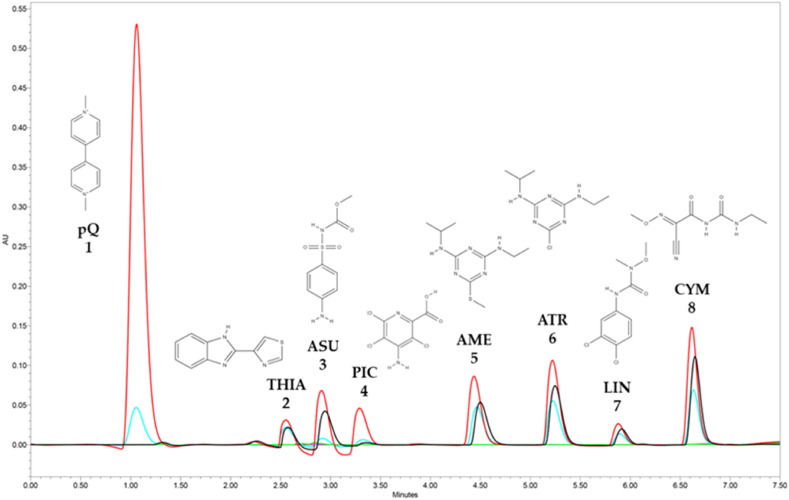
Typical UHPLC-PDA chromatograms of the separation of the selected pesticides (1, 50 mg L^−1^ and 2–8, 25 mg L^−1^) in MeOH (standard solution, red line), un-doped (wastewater, green line), and doped samples upon the μSPEed (blue line), or μQuEChERS-dSPE extraction (black line). Blank assay (solvent, pink line). 1 – pQ (Paraquat), 2 – THIA (Thiabendazole), 3 – ASU (Asulam), 4 – PIC (Picloram), 5 – AME (Ametryn), 6 – ATR (Atrazine), 7 – LIN (Linuron), 8 – CYM (Cymoxanil). (For interpretation of the references to colour in this figure legend, the reader is referred to the Web version of this article.)

Different approaches to analyse the pesticides selected for this work have been previously reported in the literature using liquid chromatography (LC), but involving very diverse conditions, time-consuming separations, and often mass detection (a literature survey of recent examples is available in Supplementary Table 1). Accordingly, the main challenge was to achieve a fast chromatographic separation coupled with UV detection capable of performing the simultaneous analysis of the selected pesticides. To achieve this, different column chemistries and temperatures, gradient compositions and flows, were thoroughly tested (data not shown) to obtain an optimal chromatographic separation (Fig. 3).

Briefly, the eight pesticides were chromatographically separated using a 7.5-min gradient composed of acidified water (0.1% FA, mobile phase A) and acetonitrile (detailed conditions indicated in Material and Methods, section 2.4). This represents an important improvement in terms of analysis time and efficient chromatographic analysis for the selected pesticides compared to the methodologies reported so far (see Supplementary Table 1). Fig. 3 also shows a comparison of the efficiency of the extraction methods developed in this work, μSPEed and μQuEChERS-dSPE. Overall, μSPEed recoveries are better than those obtained using μQuEChERS-dSPE. The exceptions are pQ and PIC, whose extractions are not as satisfactory as the remaining pesticides. In fact, pQ was not retained at all, and PIC is hardly distinguishable from the baseline upon μQuEChERS-dSPE.

### 3.4 Validation of the developed methodologies

Following the optimization of the μSPEed procedure and chromatographic analysis, both methodologies, μSPEed/UHPLC-PDA and μQuEChERS-dSPE/UHPLC-PDA, were evaluated in terms of selectivity, linearity, LODs, LOQs, matrix effect, trueness on real samples, and precision (intra-day and inter-day). The selectivity of both methodologies was good because no impurities were observed in the retention times (RTs) of the target analytes (Fig. 3). In agreement with this, no matrix effect was also observed. The data regarding the validation of both methodologies are available in Table 2. As referred to above, pQ and PIC extractions using μQuEChERS-dSPE were not satisfactory, and so these pesticides were not included in the validation of the corresponding analytical methodology. Overall, good validation features were obtained (r^2^ > 0.9921), LDR between 0.5 and 75.0 mg L^−1^ (μSPEed) and 1.0–200.0 mg L^−1^ (μQuEChERS), and LODs and LOQs of 0.02–0.05 and 0.08–0.17 mg L^−1^, respectively.

Validation also retrieved good results in terms of recoveries and intra- and inter-day precisions for both methodologies, except, as previously mentioned, for pQ and PIC, which were not considered in the μQuEChERS-dSPE/UHPLC-PDA methodology. As presented in Table 3, the recovery ranged between 66.1 ± 6.3% for CYM and 97.3 ± 10.2% for THIA, both using the *μSPEed* extraction approach. Regarding precision, all results obtained were below 15%.

**Table 3:** Recovery and precision data obtained for the selected pesticides employing both μSPEed and μQuEChERS-dSPE, in combination with UPLC-PDA.

#	MT^a^	Recovery ± SD^b^	Spiking Levels^c^	Precision^d^	
				Intra-day	Inter-day
pQ	*μSPEed*	87.9 ± 11.9	5	13.9	13.7
			50	9.8	13.3
			75	10.0	12.0
	*μQuEChERS*	–	–	–	–
THIA	*μSPEed*	97.3 ± 10.2	1	3.2	9.0
			25	8.5	11.5
			50	4.6	11.2
	*μQuEChERS*	76.5 ± 10.5	5	6.7	6.2
			25	2.6	7.8
			100	4.1	5.6
ASU	*μSPEed*	72.4 ± 9.1	1	2.2	5.7
			25	5.4	10.2
			50	5.7	12.0
	*μQuEChERS*	91.2 ± 7.5	5	5.6	5.9
			25	2.4	6.3
			100	3.6	4.5
PIC	*μSPEed*	91.3 ± 9.9	1	4.5	13.6
			25	4.5	14.1
			50	4.7	13.1
	*μQuEChERS*	–	–	–	–
AME	*μSPEed*	84.7 ± 9.5	1	4.6	13.3
			25	4.2	13.6
			50	4.8	9.9
	*μQuEChERS*	88.0 ± 6.5	5	4.5	8.4
			25	3.3	4.9
			100	4.1	4.5
ATR	*μSPEed*	85.2 ± 9.0	1	5.0	14.7
			25	9.9	10.1
			50	4.1	9.3
	*μQuEChERS*	84.0 ± 7.8	5	5.8	6.4
			25	12.0	6.7
			100	4.1	4.6
LIN	*μSPEed*	69.5 ± 7.8	1	3.9	14.0
			25	5.2	10.5
			50	4.7	14.1
	*μQuEChERS*	73.2 ± 8.4	5	6.6	7.6
			25	2.5	6.6
			100	5.8	4.7
CYM	*μSPEed*	66.1 ± 6.3	1	4.8	14.2
			25	7.4	12.1
			50	4.1	8.3
	*μQuEChERS*	83.0 ± 12.6	5	6.2	7.4
			25	2.4	4.4
			100	5.8	3.8

a Microextraction technique.

b Average of the recovery values at 25 mg L^−1^of each compound with standard solutions, except for pQ using μSPEed, in which the concentration was 50 mg L^−1^.

c Spiking levels in mg L^−1^.

d Precision values in relative standard deviation (% RSD).

### 3.5 Comparison of the developed methodologies for the analysis of the selected pesticides in wastewater samples

**Fig. 4:**
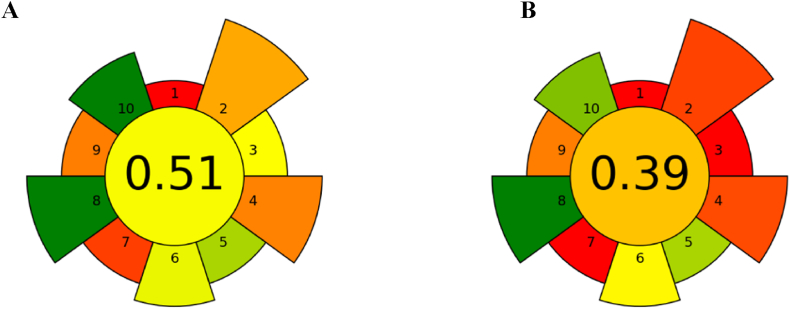
Evaluation of the greener profile of the μSPEed (A) and μQuEChERS (B) sample extraction procedures optimized in this work using the AGREEprep metric proposed by Pena-Pereira, Tobiszewski, Wojnowski and Psillakis [31].

In this work, two microextraction techniques, μSPEed and μQuEChERS-dSPE, were optimized for the extraction of eight pesticides from wastewaters. Both approaches were coupled to a fast UHPLC-PDA chromatographic analysis and validated. The corresponding analytical performances are shown in Table 2, Table 3 Overall, the results obtained are very similar between the extraction approaches. LODs and LOQs are lower in μQuEChERS-dSPE for THIA, ASU, and AME, while for LIN they are lower in μSPEed (Table 2). It is very important to emphasise, however, that all eight pesticides present in the wastewater samples could be extracted and analyzed with the μSPEed technique, whereas only six were determined with μQuEChERS-dSPE. In this case, pQ and PIC were not satisfactorily recovered from the solution under the extraction conditions used. Therefore, μSPEed seems to have a higher application range than μQuEChERS-dSPE, at least for the determination of the pesticides selected in this work. There are additional features that should also be considered when comparing these extraction procedures. The μSPEed procedure is considerably faster and less laborious than the μQuEChERS-dSPE (7.5 min vs 30 min per sample). However, several samples can be processed simultaneously using the μQuEChERS-dSPE approach, enabling time optimization that depends on the capacities of the apparatus involved (e.g., centrifuges and N_2_ stream flows). Additionally, in this work, μSPEed was operated by an electronic syringe, enabling better control and reproducibility of the experimental procedure. Further advantages include the low amounts of sorbent and solvents involved, while large volumes of samples can be repeatedly loaded into the cartridge, up to 10 mL in each loading cycle. This allows for a high concentration factor for the retained analytes. In turn, μQuEChERS is a cheaper salting-out technique combined with an efficient clean-up step by dSPE that only requires ordinary labware and instruments available in most laboratories. The μQuEChERS method, like μSPEed, also has the advantage of using low volumes of solvents. However, it is difficult to automate and therefore labour-intensive, and can also lead to the formation of emulsions in solution [30]. Furthermore, the amount of waste and labware produced by μQuEChERS is still considerably large when compared with μSPEed. The greener profiles of the proposed extraction procedures were further evaluated using the AGREEprep metric proposed by Pena-Pereira et al. [31] for evaluating the environmental impact of sample preparation methods. The results obtained, in the form of AGREEprep pictograms (Fig. 4A and B) confirm the greener profile of both extraction approaches, although the value obtained for the μSPEed approach (0.51) is considerably higher than the one obtained for μQuEChERS (0.39).

Overall, the analytical performance obtained in this work using both extraction procedures is within the range of the MRLs allowed by EU legislation for each of the selected pesticides (Table 1), with the advantage of not requiring mass detection. Additionally, a literature survey of the methods reported in the last 5 years for the analysis of the selected pesticides in water samples shows that both μSPEed and μQuEChERS are reliable alternatives to reported extraction approaches (Table 4). Despite some of the methodologies reported being able to attain lower LODs than the ones achieved in this work, they present several drawbacks. This includes, for instance, the use of MS detection ([[32], [33], [34], [35], [36]]) which is more expensive, complex, and time-consuming, online extraction systems ([34,37,38]), which can be difficult to implement in routine analysis, or organic solvents usage ([39,40]), which should be avoided to mitigate the impact on the environment of the wastes generated. For these reasons, and also considering that the extraction approaches optimized here are commercially available and readily compatible with basic LC configurations available in most analytical laboratories, the proposed μSPEed and μQuEChERS procedures combined with LC-UV analysis constitute promising alternatives for the selected pesticides in this work. Moreover, considering the wide compatibility of μQuEChERS and μSPEed sorbent chemistries available, many other pesticides are potentially eligible for the application of similar protocols to the one proposed in the work.

**Table 4:** Recent literature regarding the analysis of the pesticides studied in this work in water samples.

#	Sample	Extraction approach	Analytical method	Validation parameters		Ref
				LODs μg L^−1^	Recoveries (%)	
pQ	Drinking and surface water	On-line filtration (Si cartridge)	LC-UV	0.02	30.0–102.0	[37]
	Tap and mountain water	SPE (TiO_2_ nanotubes)	CE-UV	1.95	84.1–85.6	[41]
	Surface, tap, and irrigation ditchwater	LLE (IL)	LC-MS/MS	8.0	92.3–95.1	[32]
THIA	Milli-Q, mineral and run-off water samples	HF-LPME	HPLC-FD	0.04	–	[42]
	Wastewater sample	dSPE	LC-DAD	2.6	92.9–103.9.0	[43]
THIA	River water	HF-LPME	LC-MS/MS	0.004	–	[33]
AME				0.010	–	
ASU	Ground, tap, and river water	On-line cartridge (C18)	HPLC-UV	0.2	91.5	[38]
	Tap water	SPE (C18)	1- MEKC-UV 2- MEKC-ED	1–500.0 2–400.0	1–86.0 2–88.0	[44]
PIC	Stream water samples	LLE (diethyl ether)	RPLC-UV	0.5–2.0	92.0	[39]
	River, lake, and seawater samples	extraction discs; DCM:EtAc elution	GC-FTD	0.05	16.9–52.4	[40]
	Drinking and river water	SPE (SDB-1 PS-DVB)	HPLC-DAD	0.1	99.0–105.0	[45]
AME	Seawater sample	SPE (autosampler)	UPLC-MS/MS	0.017	86.8	[34]
ATR				0.019	81.0	
AME	River, lake, and underground water	SALLE	HPLC-DAD	0.03	79.9–102	[46]
ATR				0.02	74.6–101.0	
AME	Environmental water samples	LLME	Sweeping-MEKC-UV	0.07	93.2–112	[47]
ATR				0.21	87.4–110.0	
LIN	Tap and wastewater	IL-VALLME	HPLC-DAD	2.3	94.0–96.0	[48]
	Tap and river water sample	SPE	HPLC-DAD	0.012	67.3–69.6	[49]
	Tap water samples	SPE (CSAC)	HPLC-DAD	0.039	58.2–63.5	[50]
CYM	Drinking water	SPE (Strada X)	HPLC-UV	25.0	97.0–100.6	[51]
	Drinking water, surface water, and groundwater	SPE (Oasis HLB)	HPLC-MS/MS	2.8	56.5–115.3	[35]
	Ground and river water samples	SPE (MSU-1)	UPLC-QqQ-MS/MS	<0.01	91.0–95.0	[36]

ACN: Acetonitrile; ATPS: Aqueous Two-Phase System; [C_6_MIM][PF_6_]: 1-Hexyl-3-methylimidazolium Hexafluorophosphate; CE: Capillary Electrophoresis; CSAC: Activated Carbon derived from Coconut Shell; DAD: Diode-Array Detection; DCM: Dichloro-methane; dSPE: Dispersive Solid Phase Extraction; ED: Electrochemical Detector; EtAc: Ethyl Acetate; FD: Fluorescence Detection; FTD: Flame Thermionic Detection; HCl: Hydrochloric Acid; H_2_O: water; H_3_PO_4_: Phosphoric Acid; HF-LPME: Hollow Fibre Liquid Phase Extraction; HPLC: High Performance Liquid Chromatography; HOAc: Acetic Acid; IL: Ionic Liquid; K_2_HPO_4_: Dipotassium Phosphate; LC: Liquid Chromatography; LLE: liquid-liquid extraction; MEKC: Micellar Electrokinetic Chromatography; MeOH: Methanol; MS/MS: Mass Spectrometry; MSU-1: Mesoporous Silica Material; MWCNT: Multi-Walled Carbon Nanotube; MWCNT-OH: Hydroxyl functionalized Multi-Walled Carbon Nanotube; N: Nitrogen; NaCl: Sodium Chloride; PS-DVB: Styrene-Divinylbenzene Copolymer; QqQ: Triple Quadrupole; RPLC: Reversed Phase Liquid Chromatography; SALLE: Salting-out Assisted Liquid-Liquid Extraction; SPE: Solid-Phase Extraction; TiO_2_: Titanium Oxide (IV); UPLC: Ultra High Performance Liquid Chromatography; UV: Ultraviolet; VALLME: Vortex-Assisted Liquid-Liquid Microextraction; WS: Water Sample.

## 4 Conclusions

This study compared and validated two microextraction techniques, μSPEed and μQuEChERS-dSPE, followed by UHPLC-PDA analysis, for the simultaneous extraction of eight pesticides from wastewater samples. The developed methodologies are simpler, faster, and require less sample and solvent volumes than conventional methodologies, having a lower impact on the environment. The study highlights the potential of microextraction techniques for the analysis of pesticide residues in food and environmental samples. Due to its simple format, green profile, and commercially available solutions, the μSPEed/UHPLC-PDA methodology can be readily adapted to many other analytes and samples with success.

## Author contribution statement

Laura García-Cansino: Performed the experiments; Analyzed and interpreted the data; Wrote the paper.

María Ángeles García, María Luisa Marina: Conceived and designed the experiments; Analyzed and interpreted the data.

José S. Câmara: Conceived and designed the experiments; Analyzed and interpreted the data; Contributed reagents, materials, analysis tools or data.

Jorge A. M. Pereira: Conceived and designed the experiments; Performed the experiments; Analyzed and interpreted the data; Wrote the paper.

## Data availability statement

Data included in article/supp. material/referenced in article.

## Declaration of competing interest

The authors declare that they have no known competing financial interests or personal relationships that could have appeared to influence the work reported in this paper.
