# Supplementary material for: Simultaneous microextraction of pesticides from wastewater using optimized μSPEed and μQuEChERS techniques for food contamination analysis
Source: Heliyon. 2023 May 26;9(6):e16742. doi: 10.1016/j.heliyon.2023.e16742 (PMC10241853; doi:10.1016/j.heliyon.2023.e16742)
Supplement: Multimedia component 1 [file mmc1.docx]

**Supplementary Table 1.** Recent literature regarding the analysis of the pesticides studied in this work in water samples (detailed extraction procedures).

| # | Sample | Extraction approach | Sample treatment | Analytical method | Validation parameters | | Ref |
| --- | --- | --- | --- | --- | --- | --- | --- |
|  |  |  |  |  | **LODs**  **µg L^−1^** | **Recoveries (%)** |  |
| pQ | Drinking and surface water | On-line filtration (Si cartridge) | Water samples were passed through the cartridge in the enrichment side. Desorption was carried out by coupling the cartridge on-line with the analytical column. The elution was performed in the opposite direction to the sample preconcentration. | LC-UV | 0.02 | 30.0-102.0 | [1] |
|  | Tap and mountain water | SPE (TiO_2_ nanotubes) | N doped TiO_2_ nanotubes SPE cartridge were used for the extraction employing 3 mL of 1 M HCl/MeOH (70:30, v:v) as eluent. | CE-UV | 1.95 | 84.1-85.6 | [2] |
|  | Surface, tap, and irrigation ditch water | LLE (IL) | ATPS based on the mix of 5.0 mL of sample solution with 300 µL of IL and 6.5 g of salt. The resulting homogenate was centrifuged, and the IL phase was collected. | LC-MS/MS | 8.0 | 92.3-95.1 | [3] |
| THIA | Milli-Q, mineral and run-off water samples | HF-LPME | HF-LPME with a 3.0 cm polypropylene fiber filled with 1-octanol to extract 20 mL of H_2_O samples at pH 9.0 containing 20% (v/v) of NaCl for 30 min at 1440 rpm. The 10 µL extracted of the fiber were evaporated and reconstituted in 50 µL of mobile phase. | HPLC-FD | 0.02e^−2^-0.05 | 90.0-110.0 | [4] |
|  | Wastewater sample | dSPE | dSPE procedure was applied for 10 mL of water sample with 7 mg MWCNT-OH. The upper water phase was discarded, and the result was desorbed by ultrasound (10 min) by 1.0 mL of methanol with 200 μL of [C_6_MIM][PF_6_] and centrifuged for 5 min at 5000 rpm. | LC-DAD | 2.6 | 92.9–103.9.0 | [5] |
|  | River water | HF-LPME | WS was prepared by adjusting the pH to 7.0. HF-LPME based on a piece of hollow fiber cleaned, dried, saturated and loaded with 60 µL of 1-octanol. Finally, 1000 mL of WS was introduced. | LC-MS/MS | 4e^−3^ | 100.9-113.9 | [6] |
| ASU | Ground, tap, and river water | On-line cartridge (C18) | A syringe-type minicolumn containing 40 µL of C18 bonded silica was used and connected to the injector of the HPLC system. 130 µL of ACN:K_2_HPO_4_ buffer (pH 3.2) was employed as eluent. | HPLC-UV | 0.2 | 91.5 | [7] |
|  | Tap water | SPE (C18) | 15 mL of doped sample was acidified to pH 3.0, passed through a conditioned C18 cartridge, and eluted with 2 mL MeOH. The extract was evaporated and reconstituted with 2 mL H_2_O. | 1- MEKC-UV  2- MEKC-ED | 1- 500.0  2- 400.0 | 1- 86.0  2- 88.0 | [8] |
| PIC | Stream water samples | LLE (diethyl ether) | 800 mL of stream water (pH 2.0) and extracted 3 times with diethyl ether. The extracts were combined, and the organic solvent was evaporated. The resulting samples were transferred to vials with 4% acetic acid in ether and evaporated to dryness. The adsorbate was trapped on a reversed-phase sorbent (C18 SEP-PAK) as an additional clean-up procedure. 1 mL of 4% HOAc in water was added to the sample vial and allowed to stand overnight or held at 60° for 1 hr. This solution was loaded onto the sorbent. Desorption was performed with 9.0 mL of 25% HOAc in H_2_O. | RPLC-UV | 0.5-2.0 | 92.0 | [9] |
|  | River, lake, and sea water samples | extraction discs; DCM:EtAc elution | To 1 L of spiked sample (pH 3.0) MeOH was added to facilitate extraction. The sample was passed through extraction discs at a flow rate of 50 mL min^−1^ under vacuum. The analytes were eluted using 2x5 mL DCM:EtAc (1:1, v:v). The extract was evaporated, redissolved in 2 mL of n-hexane, and re-evaporated to 1 mL. | GC-FTD | 0.05 | 16.9-52.4 | [10] |
|  | Drinking and river wáter  (10 mL) | SPE (SDB-1 PS-DVB) | Filtered and spiked WS were passed through SDB-1 PS-DVB cartridges activated with 10 mL of acidified water. The analytes were eluted with 2 mL MeOH:ACN (1:1, v:v). The solvent was evaporated, and the extract was reconstituted with MeOH/phosphate buffer (pH 7.0) (25:85, v:v). | HPLC-DAD | 0.1 | 99.0-105.0 | [11] |
| AME | Seawater sample | SPE (autosampler) | SPE was performed with a CTC hTC PAL autosampler. 5 mL of sample was loaded at 5 mL min^−1^ onto the cartridge. The analytes were then eluted from the cartridge to the LC column with the chromatographic mobile phase. | UPLC-MS/MS | 0.017 | 86.8 | [12] |
| ATR |  |  |  |  | 0.019 | 81.0 |  |
| AME | River, lake, and underground water | SALLE | SALLE was used for herbicide extraction: 5 mL of spiked sample at pH 6.0 was mixed with 1.4 g NaCl (salting out agent) and 1 mL ACN (extraction solvent). The mixture was centrifuged, dried, ressupended in the mobile phase and injected. | HPLC-DAD | 3e^−5^ | 79.9-102 | [13] |
| ATR |  |  |  |  | 2e^−5^ | 74.6-101.0 |  |
| AME | Environmental water samples | LLME | 10 µL of H_3_PO_4_ (acceptor phase) were introduced into a 10 µL microsyringe. The fiber was immersed for 30 s to permeate its pores and then 6 mL of sample (under agitation) permeated it for 40 min for extraction. Finally, the acceptor phase was reintroduced into the microsyringe and injected into the CE. | Sweeping-MEKC-UV | 0.07 | 93.2-112 | [14] |
| ATR |  |  |  |  | 0.21 | 87.4-110.0 |  |
| LIN | Tap and wastewater | IL-VALLME | IL-VALLME was used as a microextraction technique: 10 mL of sample was mixed with NaCl, and 40 µL of an IL as extractant. Then, 2 min of shaking and 2 min of centrifugation. | HPLC-DAD | 2.3 | 94.0-96.0 | [15] |
|  | Tap and river water sample | SPE | The optimal conditions for SPE of the selected pesticides were the following: 24 mg of the T-MWCNTs for preparation SPE disk, sample volume of 500 mL at pH 5.5, 6 mL of MeOH as eluent, flow rate of sample and eluent 4 mL min^−1^. | HPLC-DAD | 0.012 | 67.3-69.6 | [16] |
|  | Tap water samples | SPE (CSAC) | SPE-CSAC method for the extraction under the following conditions: 50 mg of CSAC, sample volume of 500 mL at pH 5.5, 6 mL of DCM as eluent, flow rate of sample and eluent 3.8, and 1 mL min^−1^, respectively. | HPLC-DAD | 0.039 | 58.2-63.5 | [17] |
| CYM | Drinking water | SPE (Strada X) | WS was filtered and spiked with CYM, which was retained on a Strada X cartridge, and eluted with 2x2.0 mL of MeOH:ACN (1:1, v:v). The residue was evaporated and reconstituted in 500 µL of the mobile phase (ACN:H_2_O (30:70, v:v)). | HPLC-UV | 25.0 | 97.0-100.6 | [18] |
|  | Drinking water, surface water, and groundwater | SPE (Oasis HLB) | 500 mL of sample were pre-treated with 100 mg of sodium thiosulphate (for chlorinated drinking water) and 15 mL of ChlorAC buffer solution, followed by fortification with a standard solution. Then, 400 mL of sample at 40 mL min were passed through the Waters Oasis HLB (6 mL, 200 mg) followed of 1 mL of H_2_O to clean it. The pesticides were eluted from the cartridge with 4+2mL of MeOH:acetone (3:2) at 2 mL min^−1^. | HPLC-MS/MS | 2.8 | 56.5-115.3 | [19] |
|  | Ground and river water samples | SPE (MSU-1) | The MSU-1 cartridge was used for the extraction. It was conditioned and 100 mL of spiked sample pH 3.5 was run through. The cartridge was then washed with 5 mL H_2_O, and dried with N_2_. The analytes were eluted with 5 mL of ACN. The extract was evaporated and reconstituted in ACN:H_2_O (20:80, v:v). | UPLC-QqQ-MS/MS | <0.01 | 91.0-95.0 | [20] |

ACN: Acetonitrile; ATPS: Aqueous Two-Phase System; [C_6_MIM][PF_6_]: 1-Hexyl-3-methylimidazolium Hexafluorophosphate; CE: Capillary Electrophoresis; CSAC: Activated Carbon derived from Coconut Shell; DAD: Diode-Array Detection; DCM: Dichloro-methane; dSPE: Dispersive Solid Phase Extraction; ED: Electrochemical Detector; EtAc: Ethyl Acetate; FD: Fluorescence Detection; FTD: Flame Thermionic Detection; HCl: Hydrochloric Acid; H_2_O: water; H_3_PO_4_: Phosphoric Acid; HF-LPME: Hollow Fibre Liquid Phase Extraction; HPLC: High Performance Liquid Chromatography; HOAc: Acetic Acid; IL: Ionic Liquid; K_2_HPO_4_: Dipotassium Phosphate; LC: Liquid Chromatography; LLE: liquid-liquid extraction; MEKC: Micellar Electrokinetic Chromatography; MeOH: Methanol; MS/MS: Mass Spectrometry; MSU-1: Mesoporous Silica Material; MWCNT: Multi-Walled Carbon Nanotube; MWCNT-OH: Hydroxyl functionalized Multi-Walled Carbon Nanotube; N: Nitrogen; NaCl: Sodium Chloride; PS-DVB: Styrene-Divinylbenzene Copolymer; QqQ: Triple Quadrupole; RPLC: Reversed Phase Liquid Chromatography; SALLE: Salting-out Assisted Liquid-Liquid Extraction; SPE: Solid-Phase Extraction; TiO_2_: Titanium Oxide (IV); UPLC: Ultra High Performance Liquid Chromatography; UV: Ultraviolet; VALLME: Vortex-Assisted Liquid-Liquid Microextraction; WS: Water Sample.

References

1. Ibanez, M.; Pico, Y.; Manes, J. On-line liquid chromatographic trace enrichment and highperformance liquid chromatographic determination of diquat, paraquat and difenzoquat in water. *J. Chromatogr. A* **1996**, *728*, 325-331, doi:Doi 10.1016/0021-9673(95)00902-7.

2. Zhou, Q.X.; Mao, J.L.; Xiao, J.P.; Xie, G.H. Determination of paraquat and diquat preconcentrated with N doped TiO2 nanotubes solid phase extraction cartridge prior to capillary electrophoresis. *Analytical Methods* **2010**, *2*, 1063-1068, doi:10.1039/c0ay00250j.

3. Sha, O.; Wang, Y.; Chen, X.B.; Chen, J.; Chen, L. Determination of Paraquat in Environmental Water by Ionic Liquid-Based Liquid Phase Extraction with Direct Injection for HPLC. *J. Anal. Chem.* **2018**, *73*, 862-868, doi:10.1134/S1061934818090083.

4. Asensio-Ramos, M.; Hernandez-Borges, J.; Gonzalez-Hernandez, G.; Rodriguez-Delgado, M.A. Hollow-fiber liquid-phase microextraction for the determination of pesticides and metabolites in soils and water samples using HPLC and fluorescence detection. *Electrophoresis* **2012**, *33*, 2184-2191, doi:10.1002/elps.201200138.

5. Xu, X.; Long, N.; Lv, J.N.; Wang, L.L.; Zhang, M.H.; Qi, X.Y.; Zhang, L. Functionalized Multiwalled Carbon Nanotube as Dispersive Solid-Phase Extraction Materials Combined with High-Performance Liquid Chromatography for Thiabendazole Analysis in Environmental and Food Samples. *Food Analytical Methods* **2016**, *9*, 30-37, doi:10.1007/s12161-015-0167-x.

6. Salvatierra-Stamp, V.; Muniz-Valencia, R.; Jurado, J.M.; Ceballos-Magana, S.G. Hollow fiber liquid phase microextraction combined with liquid chromatography-tandem mass spectrometry for the analysis of emerging contaminants in water samples. *Microchem. J.* **2018**, *140*, 87-95, doi:10.1016/j.microc.2018.04.012.

7. Suzuki, T.; Yaguchi, K.; Kano, I. Screening Methods for Asulam, Oxine-Copper and Thiram in Water by High-Performance Liquid-Chromatography after Enrichment with a Minicolumn. *J. Chromatogr.* **1993**, *643*, 173-179, doi:Doi 10.1016/0021-9673(93)80550-R.

8. Chicharro, M.; Zapardiel, A.; Bermejo, E.; Sanchez, A. Simultaneous UV and electrochemical determination of the herbicide asulam in tap water samples by micellar electrokinetic capillary chromatography. *Anal. Chim. Acta* **2002**, *469*, 243-252, doi:10.1016/S0003-2670(02)00724-9.

9. Wells, M.J.M.; Michael, J.L.; Neary, D.G. Determination of Picloram in Soil and Water by Reversed-Phase Liquid-Chromatography. *Arch. Environ. Contam. Toxicol.* **1984**, *13*, 231-235, doi:Doi 10.1007/Bf01055881.

10. Albanis, T.A.; Hela, D.G. Multi-Residue Pesticide Analysis in Environmental Water Samples Using Solid-Phase Extraction Discs and Gas-Chromatography with Flame Thermionic and Mass-Selective Detection. *J. Chromatogr. A* **1995**, *707*, 283-292, doi:Doi 10.1016/0021-9673(95)00334-J.

11. Guenu, S.; Hennion, M.C. Evaluation of new polymeric sorbents with high specific surface areas using an on-line solid-phase extraction liquid chromatographic system for the trace-level determination of polar pesticides. *J. Chromatogr. A* **1996**, *737*, 15-24, doi:Doi 10.1016/0021-9673(96)00021-0.

12. Rodriguez-Gonzalez, N.; Beceiro-Gonzalez, E.; Gonzalez-Castro, M.J.; Alpendurada, M.F. On-line solid-phase extraction method for determination of triazine herbicides and degradation products in seawater by ultra-pressure liquid chromatography-tandem mass spectrometry. *J. Chromatogr. A* **2016**, *1470*, 33-41, doi:10.1016/j.chroma.2016.10.007.

13. Teju, E.; Tadesse, B.; Megersa, N. Salting-out-assisted liquid-liquid extraction for the preconcentration and quantitative determination of eight herbicide residues simultaneously in different water samples with high-performance liquid chromatography. *Sep. Sci. Technol.* **2021**, *56*, 719-729, doi:10.1080/01496395.2016.1276596.

14. Yang, Q.; Chen, B.; He, M.; Hu, B. Sensitive determination of seven triazine herbicide in honey, tomato and environmental water samples by hollow fiber based liquid-liquid-liquid microextraction combined with sweeping micellar electrokinetic capillary chromatography. *Talanta* **2018**, *186*, 88-96, doi:10.1016/j.talanta.2018.04.012.

15. Trtic-Petrovic, T.M.; Dimitrijevic, A. Vortex-assisted ionic liquid based liquid-liquid microextraction of selected pesticides from a manufacturing wastewater sample. *Central European Journal of Chemistry* **2014**, *12*, 98-106, doi:10.2478/s11532-013-0352-y.

16. Zdolsek, N.; Kumric, K.; Kalijadis, A.; Trtic-Petrovic, T. Solid-phase extraction disk based on multiwalled carbon nanotubes for the enrichment of targeted pesticides from aqueous samples. *J. Sep. Sci.* **2017**, *40*, 1564-1571, doi:10.1002/jssc.201600957.

17. Kumric, K.; Vujasin, R.; Egeric, M.; Petrovic, D.; Devecerski, A.; Matovic, L. Coconut Shell Activated Carbon as Solid-Phase Extraction Adsorbent for Preconcentration of Selected Pesticides from Water Samples. *Water Air and Soil Pollution* **2019**, *230*, doi:10.1007/s11270-019-4359-7.

18. Fidente, P.; Di Giovanni, C.; Seccia, S.; Morrica, P. Determination of cymoxanil in drinking water and soil using high-performance liquid chromatography. *Biomed. Chromatogr.* **2005**, *19*, 766-770, doi:10.1002/bmc.513.

19. Rodrigues, A.M.; Ferreira, V.; Cardoso, V.V.; Ferreira, E.; Benoliel, M.J. Determination of several pesticides in water by solid-phase extraction, liquid chromatography and electrospray tandem mass spectrometry. *J. Chromatogr. A* **2007**, *1150*, 267-278, doi:10.1016/j.chroma.2006.09.083.

20. Kharbouche, L.; Gil Garcia, M.D.; Lozano, A.; Hamaizi, H.; Galera, M.M. Solid phase extraction of pesticides from environmental waters using an MSU-1 mesoporous material and determination by UPLC-MS/MS. *Talanta* **2019**, *199*, 612-619, doi:10.1016/j.talanta.2019.02.092.
